# The Impact of Outpatient Chemotherapy-Related Adverse Events on the Quality of Life of Breast Cancer Patients

**DOI:** 10.1371/journal.pone.0124169

**Published:** 2015-04-27

**Authors:** Tomoya Tachi, Hitomi Teramachi, Kazuhide Tanaka, Shoko Asano, Tomohiro Osawa, Azusa Kawashima, Masahiro Yasuda, Takashi Mizui, Takumi Nakada, Yoshihiro Noguchi, Teruo Tsuchiya, Chitoshi Goto

**Affiliations:** 1 Laboratory of Clinical Pharmacy, Gifu Pharmaceutical University, Gifu, Japan; 2 Department of Pharmacy, Gifu Municipal Hospital, Gifu, Japan; 3 Department of Breast Surgery, Gifu Municipal Hospital, Gifu, Japan; 4 Community Health Support and Research Center, Gifu, Japan; The First Affiliated Hospital with Nanjing Medical University, CHINA

## Abstract

The objective of our study was to clarify the impact of adverse events associated with the initial course of outpatient chemotherapy on the quality of life of breast cancer patients. We conducted a survey to assess the quality of life in 48 breast cancer patients before and after receiving their first course of outpatient chemotherapy at Gifu Municipal Hospital. Patients completed the European Quality of Life 5 Dimensions and Quality of Life Questionnaire for Cancer Patients Treated with Anticancer Drugs before and after 1 course of outpatient chemotherapy. European Quality of Life 5 Dimensions utility value and Quality of Life Questionnaire for Cancer Patients Treated with Anticancer Drugs total score decreased significantly after chemotherapy (*p*<0.001 and *p* = 0.018, respectively). The mean scores for the activity, physical condition, and psychological condition subscales of the Quality of Life Questionnaire for Cancer Patients Treated with Anticancer Drugs decreased significantly after chemotherapy (*p* = 0.003, *p*<0.001, and *p* = 0.032, respectively), whereas the social relationships score increased significantly (*p*<0.001). Furthermore, in the evaluation of quality of life according to individual adverse events, the decrease in quality of life after chemotherapy in terms of the European Quality of Life 5 Dimensions utility value and the Quality of Life Questionnaire for Cancer Patients Treated with Anticancer Drugs total score was greater in anorexic patients than in non-anorexic patients (*p* = 0.009 and *p*<0.001, respectively). This suggests that anorexia greatly reduces quality of life. Our findings reveal that anticancer drug-related adverse events, particularly anorexia, reduce overall quality of life following the first course of outpatient chemotherapy in current breast cancer patients. These findings are extremely useful and important in understanding the impact of anticancer drug-related adverse events on quality of life.

## Introduction

Anticancer drug therapy has recently shifted from an inpatient to an outpatient setting, and outpatient chemotherapy involving the injection of anticancer drugs during hospital visits is being widely practiced. The reason behind this is the development of supportive therapy and drugs that do not require hospitalization and changes in the health care environment to reduce medical costs [1,2]. Anticancer drug therapy generally has a high incidence of adverse events, which have been the subject of several reports [3,4]. The time of onset of adverse events varies greatly according to the anticancer drug, and adverse events may occur in the early stages of anticancer drug therapy. Since adverse events associated with outpatient chemotherapy, as opposed to inpatient anticancer drug therapy, directly influence home life and work, they may cause changes in patient quality of life (QOL). Therefore, it is becoming increasingly important to understand the QOL of patients receiving outpatient chemotherapy, especially given the current shift in anticancer drug therapy from the hospital to the home. Furthermore, the incidence of breast cancer has increased in recent years, and although the majority of patients are in their late 50s, the number of women developing breast cancer in their late 20s is increasing [5,6]. Women who develop breast cancer at a relatively young age may receive outpatient chemotherapy while continuing to work, which may be negatively affected by deterioration in the QOL.

Numerous QOL assessments have been performed for various diseases, including breast cancer [7–17]. For breast cancer, studies have investigated the effect of stress from breast cancer diagnosis on QOL [7], the effect of mental anguish on mortality rate and QOL [8–11], effect of age on QOL [12], the relationship between surgical procedures and QOL [13,14], QOL of patients with metastatic disease [15], QOL following surgery [16], and the association between QOL and menopausal symptoms [17]. Reports have also discussed changes in QOL due to anticancer drug monotherapy and single regimens for breast cancer [18,19] as well as changes in QOL from the time of hospitalization to after discharge when treated with anticancer drug therapy [20]. However, we could find no reports examining the impact of adverse events associated with the first course of outpatient chemotherapy on QOL in breast cancer patients. Therefore, in the present study, we investigated the impact of current outpatient chemotherapy-related adverse events on QOL in breast cancer patients and investigated the association between the type of adverse event and changes in QOL after chemotherapy.

## Methods

### Subjects and treatment period

The subjects were 48 breast cancer patients who received their first course of intravenous chemotherapy at Gifu Municipal Hospital between December 2012 and November 2013. None of the patients received hormone therapy or radiation therapy. All patients received appropriate supportive therapy according to the chemotherapy treatment guidelines for breast cancer patients. We asked the subjects to answer a self-completed survey questionnaire before and after their first course of outpatient chemotherapy (day 1 of the first and second course, respectively).

### Survey items

Survey items were patient attributes, QOL, and adverse events. Patient attributes obtained from the survey comprised marital status, presence of cohabitants, current occupation, annual income, occupation before diagnosis, education, and experience as a healthcare professional. Data were also obtained from the patients’ electronic medical records and included age; TMN classification of breast cancer progression; performance status (PS); human epidermal growth factor receptor 2 (HER2), estrogen receptor (ER), and progesterone receptor (PgR) status; purpose of chemotherapy (adjuvant, neoadjuvant, or symptom relief); and treatment regimen.

QOL was assessed using the European Quality of Life 5 Dimensions (EQ-5D) [21,22] and QOL Questionnaire for Cancer Patients Treated with Anticancer Drugs (QOL-ACD) [23]. The EQ-5D is widely used in clinical studies and health status surveys targeting the general population as a comprehensive scale based on preferences to assess cardinal changes in level of health [24,25]. The questionnaire analyzes health state over 5 dimensions: mobility, personal care, usual activities, pain/discomfort, and anxiety/depression. Each dimension has 3 levels: level 1, no problems; level 2, some problems; and level 3, major problems. The health state of the individual is defined by combining the level from each of the 5 dimensions. A utility value ranging from 0 to 1 was calculated from the EQ-5D. According to the Japanese version of the utility value conversion table, 0 indicates death and 1 indicates perfect health.

The QOL-ACD is a cancer-specific scale for which the reliability and validity has been confirmed among cancer patients in Japan. QOL assessments using the QOL-ACD have also been used for breast cancer patients [16,26,27]. The QOL-ACD is composed of 4 subscales (activity, 6 items; physical condition, 6 items; psychological condition, 5 items; and social relationships, 5 items) and a “face scale” (1 item) for measuring overall QOL (total 23 items). The lowest QOL for each item is given a score of 1, whereas the best QOL is given a score of 5. Thus, the QOL-ACD gives a minimum total score of 23 points and maximum total score of 115 points. Information regarding the occurrence of adverse events up until after the first course of outpatient chemotherapy was obtained from the electronic medical records. Two pharmacists assessed the severity of adverse events based on the Common Terminology Criteria for Adverse Events v4.0 grade classification [28]. Any disagreements in adverse event grading between the 2 pharmacists were discussed until a consensus was reached. Grade 0 indicated no adverse events, whereas grade 1 or above indicated the presence of adverse events.

### Analysis and statistical processing

The one stage 0 patient was excluded from the analysis to focus on invasive cases.

The utility value calculated from the EQ-5D, proportion of patients with problems in each of the 5 EQ-5D dimensions, total score for all items in the QOL-ACD, and mean score for each QOL-ACD subscale were compared before and after chemotherapy. In addition, changes in QOL after chemotherapy were compared between patient groups stratified according to the presence or absence and type of adverse event that occurred in 10% or more of patients.

SPSS18.0J software (IBM, Armonk, NY, USA) was used for statistical processing. The paired *t*-test was used to test for differences in the utility value, QOL-ACD total score, and mean score for each subscale before and after chemotherapy. The unpaired *t*-test was used for intergroup comparisons of changes in the utility value, QOL-ACD total score, and mean score for each subscale after chemotherapy according to the presence or absence of individual adverse events. Fisher’s exact test was used for the intergroup comparison of the proportion of patients whose scores for the 5 dimensions of the EQ-5D worsened after chemotherapy according to the presence or absence of individual adverse events. A *p*-value of < 0.05 was considered statistically significant.

### Ethics statement

The study was approved by the Ethical Review Board of Gifu Municipal Hospital and Bioethics Committee of Gifu Pharmaceutical University. Subjects were also given sufficient explanation of the study in writing, and the provided written informed consent to participate.

## Results

### Patient attributes

Table 1 shows patient attributes. All patients were women, with a mean age of 59.6 years. Most patients (43.8%) had stage 2 disease, and 91.7% of patients had a PS of 0. HER2, ER, and PgR expression were positive in 79.2%, 66.7%, and 18.8% of patients, respectively. Adjuvant, neoadjuvant and symptom relief therapy were administered to 53.2%, 36.2%, and 10.6% of patients, respectively. The majority of patients (36.4%) were administered a regimen of epirubicin plus cyclophosphamide. In addition, 81.3% of patients were married, and 97.3% of patients lived with another individual. Patients currently employed accounted for 39.6% of patients, whereas 60.4% of patients had worked prior to being diagnosed with breast cancer.

**Table 1:** Patient attributes.

		Mean ± Standard deviation	
Age (year)		59.6 ± 12.2	
		*n*	%
Stage			
	1	15	31.9
	2	21	44.7
	3	5	10.6
	4	6	12.8
PS			
	0	43	91.5
	1	1	2.1
	2	3	6.4
HER2			
	(+)	38	80.9
	(-)	9	19.1
ER			
	(+)	31	66.0
	(-)	8	17.0
	(±)	8	17.0
PgR			
	(+)	8	17.0
	(-)	24	51.1
	(±)	15	31.9
Purpose of chemotherapy			
	Adjuvant	25	53.2
	Neoadjuvant	17	36.2
	Symptom relief	5	10.6
Regimen			
	EC (every 3w)	17	36.2
	TRZ+nabPTX (every 3w)	5	10.6
	TC (every 3w)	5	10.6
	3w-nabPTX (every 3w)	4	8.5
	LPR (every 4w)	3	6.4
	FUL (every 4w)	3	6.4
	CMF (every 3w)	3	6.4
	3w-TRZ+CMF (every 3w)	3	6.4
	TRZ (every 3w)	2	4.3
	3w-TRZ+DOC (every 3w)	2	4.3
Marital status			
	Married	39	83.0
	Unmarried	8	17.0
Cohabitants			
	Yes	46	97.9
	No	1	2.1
Current occupation			
	Unemployed, housewife, student	28	59.6
	Temporary employment, part-time employment	10	21.3
	Company employee, self-employed	6	12.8
	Public official	0	0.0
	Other	3	6.4
Occupation before diagnosis			
	Unemployed, housewife, student	19	40.4
	Temporary employment, part-time employment	17	36.2
	Company employee, self-employed	8	17.0
	Public official	0	0.0
	Other	3	6.4
Education			
	Junior school graduate	12	25.5
	High school graduate	18	38.3
	Vocational school, junior college, technical school graduate	14	29.8
	University graduate, postgraduate	3	6.4
Experience as a healthcare professional			
	Currently a healthcare professional	0	0.0
	Was a healthcare professional in the past	2	4.3
	Was not a healthcare professional in the past	45	95.7

PS: performance status, HER2: human epidermal growth factor receptor type 2, ER: estrogen receptor, PgR: progesterone receptor, EC: epirubicin/cyclophosphamide、TC: docetaxel/cyclophosphamide, TRZ: trastuzumab, DOC: docetaxel, LPR: leuprorelin, FUL: fulvestrant, CMF: cyclophosphamide/methotrexate/fluorouracil, nabPTX: nab-paclitaxel, 3w: 3 weeks, 4w: 4 weeks.

### QOL assessment

Table 2 shows the changes in EQ-5D after chemotherapy. The utility value decreased significantly after treatment (before chemotherapy, 0.84 ± 0.17; after chemotherapy, 0.73 ± 0.18; *p*<0.001), and the proportion of patients who responded that they had problems in the mobility and usual activities dimensions of the EQ-5D increased significantly after chemotherapy (*p* = 0.024 and *p* = 0.003, respectively). Table 3 shows the changes in QOL-ACD after chemotherapy. The QOL-ACD total score (before chemotherapy, 88.6 ± 11.1; after chemotherapy, 84.6 ± 14.0; *p* = 0.018) and mean scores for the QOL-ACD subscales of activity (before chemotherapy, 4.57 ± 0.79; after chemotherapy, 4.15 ± 1.00; *p* = 0.003), physical condition (before chemotherapy, 4.34 ± 0.52; after chemotherapy, 3.95 ± 0.71; *p*<0.001), and psychological condition (before chemotherapy, 3.99 ± 0.66; after chemotherapy, 3.76 ± 0.80; *p* = 0.032) decreased significantly after treatment. On the other hand, the mean score for social relationships increased significantly after treatment (before chemotherapy, 2.36 ± 0.74; after chemotherapy, 2.72 ± 0.76; *p*<0.001).

**Table 2:** Comparison of the EQ-5D utility value and 5 dimensions before and after 1 course of outpatient chemotherapy.

		Before CT	After CT	*p*
Utility value		0.84±0.17	0.73±0.18	<0.001^*^
Dimensions		Some or major problems(%)		
	Mobility	10.6	29.8	0.024^*^
	Personal care	6.4	12.8	0.486
	Usual activities	10.6	38.3	0.003^*^
	Pain/discomfort	42.6	51.1	0.535
	Anxiety/depression	40.4	53.2	0.301

CT: chemotherapy,

*p<0.05

**Table 3:** Comparison of the QOL-ACD total score and mean score for each subscale before and after 1 course of outpatient chemotherapy.

		Before CT	After CT	*p*
Total score		88.6±11.1	84.6±14.0	0.018^*^
Average score of subscales				
	Activity	4.57±0.79	4.15±1.00	0.003^*^
	Physical condition	4.34±0.52	3.95±0.71	<0.001^*^
	Psychological condition	3.99±0.66	3.76±0.80	0.032^*^
	Social relationships	2.36±0.74	2.72±0.76	<0.001^*^

CT: chemotherapy, mean±standard deviation,

*p<0.05

### Relationship between QOL and adverse events

Table 4 shows the classification of individual adverse events according to grade. Tables 5 and 6 show the results of the EQ-5D QOL assessment according to the presence or absence of individual adverse events. The degree of change in the utility value after treatment was significant for patients with anorexia (*p* = 0.009). Moreover, changes in the utility value after treatment were significant for the adverse events of alopecia (*p* = 0.008), constipation (*p* = 0.019), malaise (*p* = 0.028), and anorexia (*p* = 0.030) in the usual activities dimension and the adverse event of alopecia (*p* = 0.018) in the pain/discomfort dimension. Tables 7 and 8 show the QOL-ACD results according to the presence or absence of individual adverse events. The degree of change in the total QOL-ACD score after treatment was only significant for the adverse event of alopecia (*p*<0.001). The adverse events of fever (*p* = 0.017) and alopecia (*p* = 0.018) in the activity scale; nausea (*p* = 0.014), oral pain (*p* = 0.026), dysgeusia (*p* = 0.026), and anorexia (*p*<0.001) in the physical condition scale; anorexia (*p* = 0.007) in the psychological condition scale; and anemia (*p* = 0.020) in the social relationships scale were associated with significant changes in subscale mean scores after treatment. However, the decrease in the mean score for physical condition after treatment was greater in the group without peripheral sensory neuropathy than in the group with peripheral neuropathy. By contrast, the increase in the mean score for social relationships after treatment was larger in patients with anemia than in those without anemia. Table 9 shows a comparison of the EQ-5D utility value and QOL-ACD total score according to the presence or absence of anorexia in patients stratified by stage, purpose of chemotherapy and age. The degree of change in the total QOL-ACD score after treatment was significant in patients with stage 1 (*p* = 0.004) and stage 2–4 (*p* = 0.006) disease. The degree of change in the utility value and total QOL-ACD score after treatment was significant in patients receiving neoadjuvant therapy (both, *p*<0.001). The degree of change in the utility value and total QOL-ACD score after treatment was significant in patients ≥50 years of age (respectively, *p* = 0.021 and *p* = 0.005), whereas that of the total QOL-ACD score was significant in patients <49 years of age (*p*<0.001).

**Table 4:** Occurrence of individual adverse events.

Adverse events (*n*)	Grade 0	Grade 1	Grade 2	Grade 3
Alopecia	16 (34.0%)	12 (25.5%)	19 (40.4%)	0 (0%)
Nail ridging	42 (89.4%)	5 (10.5%)	0 (0%)	0 0%)
Dry skin	40 (85.1%)	7 (14.9%)	0 (0%)	0 (0%)
Nausea	35 (74.5%)	9 (19.1%)	3 (6.4%)	0 (0%)
Constipation	31 (66.0%)	12 (25.5%)	3 (6.4%)	1 (2.1%)
Mucositis oral	35 (74.5%)	10 (21.3%)	2 (4.3%)	0 (0%)
Oral pain	42 (89.4%)	5 (10.6%)	0 (0%)	0 0%)
Peripheral sensory neuropathy	38 (80.9%)	9 (19.1%)	0 (0%)	0 (0%)
Dysgeusia	39 (83.0%)	3 (6.4%)	5 (10.6%)	0 (0%)
Malaise	21 (44.7%)	19 (40.4%)	7 (14.9%)	0 0%)
Fever	40 (85.1%)	7 (14.9%)	0 (0%)	0 (0%)
White blood cell decreased	37 (78.7%)	8 (17.0%)	2 (4.3%)	0 (0%)
Anemia	29 (61.7%)	18 (38.3%)	0 (0%)	0 0%)
Anorexia	27 (57.4%)	9 (19.1%)	11 (23.4%)	0 (0%)

**Table 5:** Comparison of the EQ-5D utility value according to the presence or absence of individual adverse events.

Adverse events			Utility value			
	Grade	*N*	Before CT (A)	After CT (B)	Difference (B-A)	*p*
					
Alopecia						0.224
	0	16	0.83±0.19	0.76±0.18	-0.07±0.14	
	1–3	31	0.84±0.15	0.71±0.19	-0.13±0.18	
Nail ridging						0.541
	0	42	0.84±0.17	0.73±0.19	-0.11±0.17	
	1–3	5	0.84±0.15	0.68±0.15	-0.16±0.15	
Dry skin						0.802
	0	40	0.85±0.16	0.74±0.18	-0.11±0.18	
	1–3	7	0.76±0.20	0.64±0.16	-0.13±0.13	
Nausea						0.646
	0	35	0.83±0.17	0.72±0.20	-0.10±0.18	
	1–3	12	0.87±0.16	0.74±0.12	-0.13±0.14	
Constipation						0.239
	0	31	0.85±0.14	0.76±0.14	-0.09±0.16	
	1–3	16	0.82±0.15	0.67±0.24	-0.15±0.18	
Mucositis oral						0.237
	0	35	0.84±0.16	0.74±0.19	-0.09±0.18	
	1–3	12	0.84±0.19	0.68±0.17	-0.16±0.14	
Oral pain						0.927
	0	42	0.85±0.17	0.74±0.19	-0.11±0.18	
	1–3	5	0.78±0.13	0.66±0.15	-0.12±0.12	
Peripheral sensory neuropathy						0.628
	0	38	0.86±0.15	0.74±0.18	-0.12±0.18	
	1–3	9	0.76±0.21	0.67±0.18	-0.08±0.15	
Dysgeusia						0.705
	0	39	0.83±0.17	0.72±0.19	-0.12±0.18	
	1–3	8	0.86±0.15	0.77±0.12	-0.09±0.10	
Malaise						0.643
	0	21	0.87±0.16	0.78±0.14	-0.10±0.14	
	1–3	26	0.81±0.17	0.69±0.21	-0.12±0.19	
Fever						0.884
	0	40	0.85±0.16	0.74±0.19	-0.11±0.18	
	1–3	7	0.81±0.19	0.69±0.13	-0.12±0.12	
White blood cell decreased						0.059
	0	37	0.84±0.17	0.71±0.19	-0.14±0.17	
	1–3	10	0.83±0.15	0.81±0.14	-0.02±0.16	
Anemia						0.694
	0	29	0.86±0.17	0.74±0.16	-0.12±0.15	
	1–3	18	0.80±0.16	0.70±0.22	-0.10±0.21	
Anorexia						0.009*
	0	27	0.82±0.17	0.77±0.16	-0.06±0.16	
	1–3	20	0.86±0.16	0.68±0.21	-0.19±0.16	

CT: chemotherapy, mean±standard deviation,

*p<0.05

**Table 6:** Comparison of 5 dimensions according to the presence or absence of individual adverse events.

Adverse events			Mobility				Personal care				Usual activities				Pain/discomfort				Anxiety/depression			
	Grade	*n*	Some or major problems (%)		Deteriorated (%)	*p*	Some or major problems (%)		Deteriorated (%)	*p*	Some or major problems (%)		Deteriorated (%)	*p*	Some or major problems (%)		Deteriorated (%)	*p*	Some or major problems (%)		Deteriorated (%)	*p*
			Before CT	After CT			Before CT	After CT			Before CT	After CT			Before CT	After CT			Before CT	After CT		
Alopecia						1.000				1.000				0.008*				0.036*				0.739
	0	16	18.8	37.5	18.8		12.5	18.8	6.3		18.8	25.0	6.3		43.8	37.5	6.3		31.3	56.3	31.3	
	1–3	31	6.5	25.8	19.4		3.2	9.7	6.5		6.5	45.2	45.2		41.9	58.1	38.7		45.2	51.6	25.8	
Nail ridging						1.000				0.292				0.648				0.607				1.000
	0	42	11.9	31.0	19.0		7.1	11.9	4.8		11.9	38.1	31.0		40.5	47.6	26.2		40.5	52.4	28.6	
	1–3	5	0.0	20.0	20.0		0.0	20.0	20.0		0.0	40.0	40.0		60.0	80.0	40.0		40.00	60.00	20.0	
Dry skin						0.605				0.391				1.000				0.377				1.000
	0	40	10.0	27.5	17.5		5.0	10.0	5.00		10.0	37.5	32.5		40.0	45.0	25.0		37.5	50.0	27.5	
	1–3	7	14.3	42.9	28.6		14.3	28.6	14.3		14.3	42.9	28.6		57.1	85.7	42.9		57.14	71.43	28.6	
Nausea						0.412				0.560				0.158				1.000				0.269
	0	35	11.4	34.3	22.9		8.6	17.1	8.6		11.4	34.3	25.7		42.9	54.3	28.6		42.9	48.6	22.9	
	1–3	12	8.3	16.8	8.3		0.0	0.0	0.0		8.3	50.0	50.0		41.67	41.67	25.0		33.33	66.67	41.7	
Constipation						0.466				0.264				0.019*				0.096				0.168
	0	31	12.9	29.0	16.1		6.5	9.7	3.2		16.1	29.0	19.4		41.9	41.9	19.4		32.3	61.3	35.5	
	1–3	16	6.3	31.3	25.0		6.3	18.8	12.5		0.0	56.3	56.3		43.8	68.8	43.8		56.3	37.5	12.5	
Mucositis oral						0.205				0.560				1.000				0.065				0.713
	0	35	11.4	25.7	14.3		5.7	14.3	8.6		11.4	37.1	31.4		48.6	45.7	20.0		37.1	48.6	25.7	
	1–3	12	8.3	41.6	33.3		8.3	8.3	0.0		8.3	41.7	33.3		25.0	66.7	50.0		50.0	66.7	33.3	
Oral pain						1.000				0.292				0.309				0.607				0.607
	0	42	9.5	28.6	19.0		7.1	11.9	4.8		11.9	35.7	28.6		40.5	50.0	26.2		38.1	50.0	26.2	
	1–3	5	20.0	40.0	20.0		0.0	20.0	20.0		0.0	60.0	60.0		60.0	60.0	40.0		60.0	80.0	40.0	
Peripheral sensory neuropathy						1.000				1.000				1.000				0.692				1.000
	0	38	5.3	23.7	18.4		2.6	10.5	7.9		10.5	36.8	31.6		39.5	50.0	26.3		36.8	47.4	28.9	
	1–3	9	33.3	55.6	22.2		22.2	22.2	0.0		11.1	44.4	33.3		55.6	55.6	33.3		55.56	77.78	22.2	
Dysgeusia						1.000				1.000				0.246				0.413				1.000
	0	39	12.8	33.3	20.5		7.7	15.4	7.7		12.8	35.9	28.2		43.6	53.8	30.8		41.0	53.8	28.2	
	1–3	8	0.0	12.5	12.5		0.0	0.0	0.0		0.0	50.0	50.0		37.5	37.5	12.5		37.5	50.0	25.0	
Malaise						0.711				1.000				0.028*				0.744				0.746
	0	21	9.5	23.8	14.3		4.8	9.5	4.8		14.3	23.8	14.3		33.3	47.6	28.6		28.6	38.1	23.8	
	1–3	26	11.5	34.6	23.1		7.7	15.4	7.7		7.7	50.0	46.2		50.0	53.9	23.1		50.0	65.4	30.8	
Fever						0.318				1.000				0.188				1.000				1.000
	0	40	10.0	32.5	22.5		5.0	12.5	7.5		12.5	35.0	27.5		40.0	47.5	27.5		37.5	50.0	27.5	
	1–3	7	14.3	14.3	0.0		14.3	14.3	0.0		0.0	57.1	57.1		57.1	71.4	28.6		57.1	71.4	28.6	
White blood cell decreased						1.000				1.000				0.465				0.703				1.000
	0	37	13.5	32.4	18.9		8.1	16.2	8.1		10.8	43.2	35.1		40.5	54.1	29.7		40.5	56.8	27.0	
	1–3	10	0.0	20.0	20.0		0.00	0.00	0.0		10.0	20.0	20.0		50.0	40.0	20.0		40.0	40.0	20.0	
Anemia						0.716				1.000				0.202				1.000				0.315
	0	29	13.8	31.0	17.2		6.9	13.8	6.9		10.3	34.5	24.1		34.5	48.3	27.6		31.0	55.2	34.5	
	1–3	18	5.6	27.8	22.2		5.6	11.1	5.6		11.1	44.4	44.4		55.6	55.6	27.8		55.6	50.0	16.7	
Anorexia						0.142				0.567				0.030*				1.000				0.511
	0	27	14.8	25.9	11.1		7.4	11.1	3.7		18.5	29.6	18.5		44.4	55.6	25.9		40.7	40.7	22.2	
	1–3	20	5.0	35.0	30.0		5.0	15.0	10.0		0.0	50.0	50.0		40.0	45.0	30.0		40.0	70.0	35.0	

CT: chemotherapy,

*p<0.05

**Table 7:** Comparison of the QOL-ACD total score for each subscale according to the presence or absence of individual adverse events.

Adverse events	Grade	*n*	Total score			
			Before CT (A)	After CT (B)	Difference (B-A)	*p*
Alopecia						0.649
	0	16	86.25±13.91	83.31±16.20	-2.94±11.01	
	1–3	31	89.74±9.40	85.23±12.96	-4.52±11.28	
Nail ridging						0.671
	0	42	88.17±11.48	84.43±14.27	-3.74±11.40	
	1–3	5	91.80±7.43	85.80±12.85	-6.00±8.80	
Dry skin						0.795
	0	40	88.83±10.89	85.03±14.19	-3.80±11.85	
	1–3	7	87.00±13.14	82.00±13.60	-5.00±5.35	
Nausea						0.123
	0	35	87.63±10.88	85.11±15.08	-2.51±11.82	
	1–3	12	91.25±11.84	83.00±10.62	-8.25±7.48	
Constipation						0.675
	0	31	89.26±12.55	85.77±13.65	-3.48±10.02	
	1–3	16	87.19±7.78	82.25±14.82	-4.94±13.23	
Mucositis oral						0.885
	0	35	88.54±10.60	84.46±14.38	-4.09±12.30	
	1–3	12	88.58±13.01	84.92±13.43	-3.67±6.84	
Oral pain						0.309
	0	42	88.62±11.36	85.21±14.09	-3.41±11.52	
	1–3	5	88.00±9.82	79.20±13.37	-8.80±4.82	
Peripheral sensory neuropathy						0.376
	0	38	89.13±11.53	84.45±13.88	-4.68±11.05	
	1–3	9	86.11±9.36	85.11±15.34	-1.00±11.42	
Dysgeusia						0.123
	0	39	87.54±11.42	84.69±14.97	-2.85±11.49	
	1–3	8	93.50±8.37	84.00±8.45	-9.50±7.03	
Malaise						0.456
	0	21	89.10±12.91	86.48±14.74	-2.62±9.99	
	1–3	26	88.12±9.67	83.04±13.46	-5.08±11.99	
Fever						0.075
	0	40	87.48±11.39	84.70±14.34	-2.78±10.98	
	1–3	7	94.71±7.16	83.86±12.82	-10.86±9.75	
White blood cell decreased						0.229
	0	37	88.81±11.84	83.81±15.23	-5.00±11.22	
	1–3	10	87.60±8.32	87.40±7.95	-0.20±10.26	
Anemia						0.481
	0	29	89.10±11.75	86.03±14.64	-3.07±10.69	
	1–3	18	87.67±10.26	82.22±12.95	-5.44±11.87	
Anorexia						<0.001^*^
	0	27	86.40±11.76	87.48±14.65	1.07±10.25	
	1–3	20	91.45±9.71	80.65±12.35	-10.80±8.29	

CT: chemotherapy, mean±standard deviation,

*p<0.05

**Table 8:** Comparison of the QOL-ACD mean score for each subscale according to the presence or absence of individual adverse events.

Adverse events			Subscale															
	Grade	*N*	Activity				Physical condition				Psychological condition				Social relationships			
			Before CT (A)	After CT (B)	Difference (B-A)	*p*	Before CT (A)	After CT (B)	Difference (B-A)	*p*	Before CT (A)	After CT (B)	Difference (B-A)	*p*	Before CT (A)	After CT (B)	Difference (B-A)	*p*
Alopecia						0.719				0.284				0.860				0.796
	0	16	4.48±1.09	4.13±1.18	-0.35±0.79		4.16±0.69	3.91±0.77	-0.25±0.61		3.89±0.68	3.69±0.78	-0.20±0.74		2.28±0.66	2.60±0.65	0.33±0.55	
	1-3	31	4.62±0.60	4.16±0.92	-0.46±0.98		4.43±0.40	3.98±0.69	-0.45±0.60		4.04±0.66	3.80±0.83	-0.24±0.69		2.40±0.78	2.77±0.81	0.37±0.64	
Nail ridging						0.843				0.219				0.655				0.436
	0	42	4.55±0.83	4.13±1.02	-0.41±0.94		4.33±0.55	3.98±0.72	-0.35±0.60		3.97±0.69	3.76±0.81	-0.21±0.71		2.33±0.73	2.67±0.77	0.33±0.59	
	1-3	5	4.77±0.37	4.27±0.98	-0.50±0.75		4.40±0.25	3.70±0.66	-0.70±0.59		4.1±0.23	3.76±0.89	-0.36±0.71		2.56±0.86	3.12±0.58	0.56±0.79	
Dry skin						0.621				0.147				0.990				0.741
	0	40	4.60±0.78	4.15±1.00	-0.45±0.95		4.33±0.54	4.00±0.73	-0.33±0.62		4.00±0.64	3.78±0.77	-0.23±0.73		2.38±0.75	2.73±0.79	0.35±0.59	
	1-3	7	4.41±0.90	4.14±1.07	-0.26±0.70		4.38±0.46	3.69±0.50	-0.69±0.42		3.91±0.80	3.69±1.03	-0.23±0.51		2.23±0.68	2.66±0.54	0.43±0.74	
Nausea						0.485				0.017^*^				0.095				0.621
	0	35	4.54±0.84	4.18±1.07	-0.37±0.98		4.29±0.52	4.02±0.72	-0.26±0.60		3.89±0.64	3.76±0.82	-0.13±0.69		2.38±0.70	2.71±0.77	0.33±0.66	
	1-3	12	4.65±0.65	4.07±0.82	-0.58±0.71		4.49±0.53	3.75±0.67	-0.74±0.49		4.28±0.64	3.77±0.80	-0.52±0.66		2.30±0.86	2.73±0.76	0.46±0.43	
Constipation						0.111				0.787				0.338				0.522
	0	31	4.54±0.95	4.27±0.99	-0.27±0.84		4.40±0.56	4.04±0.66	-0.37±0.58		4.14±0.64	3.84±0.75	-0.30±0.69		2.29±0.71	2.61±0.73	0.32±0.53	
	1-3	16	4.63±0.31	3.91±1.02	-0.72±1.01		4.21±0.44	3.79±0.80	-0.42±0.71		3.70±0.62	3.61±0.91	-0.09±0.72		2.49±0.80	2.93±0.79	0.44±0.75	
Mucositis oral						0.793				0.959				0.602				0.875
	0	35	4.56±0.82	4.12±1.07	-0.44±1.00		4.34±0.50	3.96±0.71	-0.38±0.67		4.04±0.54	3.78±0.79	-0.26±0.73		2.31±0.77	2.68±0.78	0.37±0.63	
	1-3	12	4.60±0.72	4.24±0.62	-0.36±0.62		4.32±0.62	3.93±0.75	-0.39±0.36		3.83±0.95	3.70±0.88	-0.13±0.62		2.48±0.65	2.82±0.71	0.33±0.55	
Oral pain						0.255				0.014^*^				0.655				0.160
	0	42	4.58±0.82	4.21±0.97	-0.37±0.91		4.33±0.54	4.02±0.70	-0.31±0.59		3.94±0.66	3.73±0.80	-0.21±0.72		2.40±0.73	2.72±0.78	0.31±0.60	
	1-3	5	4.50±0.51	3.63±1.23	-0.87±0.88		4.37±0.38	3.37±0.46	-1.00±0.26		4.36±0.62	4.00±0.86	-0.36±0.50		1.96±0.82	2.68±0.61	0.72±0.64	
Peripheral sensory neuropathy						0.989				0.055				0.287				0.711
	0	38	4.64±0.79	4.22±1.00	-0.42±0.95		4.34±0.56	3.88±0.73	-0.47±0.61		4.02±0.68	3.74±0.80	-0.28±0.69		2.35±0.77	2.72±0.77	0.37±0.57	
	1-3	9	4.28±0.77	3.85±1.06	-0.43±0.80		4.32±0.35	4.28±0.52	-0.04±0.44		3.84±0.60	3.84±0.86	0.00±0.72		2.40±0.61	2.69±0.74	0.29±0.78	
Dysgeusia						0.639				0.026^*^				0.382				0.427
	0	39	4.50±0.85	4.10±1.08	-0.39±0.98		4.32±0.55	4.02±0.74	-0.30±0.59		3.95±0.66	3.77±0.78	-0.19±0.71		2.30±0.72	2.69±0.76	0.39±0.62	
	1-3	8	4.94±0.18	4.38±0.48	-0.56±0.55		4.44±0.34	3.63±0.47	-0.81±0.53		4.15±0.69	3.73±0.96	-0.43±0.68		2.63±0.83	2.83±0.80	0.20±0.56	
Malaise						0.592				0.369				0.661				0.205
	0	21	4.60±0.91	4.25±1.04	-0.34±0.82		4.33±0.63	4.03±0.72	-0.29±0.57		4.17±0.56	3.90±0.76	-0.28±0.72		2.26±0.78	2.73±0.79	0.48±0.38	
	1-3	26	4.55±0.70	4.06±0.98	-0.49±0.99		4.35±0.43	3.89±0.71	-0.46±0.64		3.84±0.71	3.65±0.84	-0.19±0.70		2.44±0.70	2.70±0.74	0.26±0.74	
Fever						0.017^*^				0.092				0.348				0.385
	0	40	4.52±0.84	4.18±1.06	-0.34±0.96		4.30±0.55	3.98±0.73	-0.32±0.60		3.95±0.69	3.77±0.79	-0.19±0.68		2.29±0.73	2.68±0.75	0.39±0.59	
	1-3	7	4.86±0.31	3.98±0.58	-0.88±0.38		4.52±0.30	3.79±0.58	-0.74±0.56		4.20±0.46	3.74±0.97	-0.46±0.83		2.77±0.69	2.94±0.79	0.17±0.73	
White blood cell decreased						0.115				0.286				0.121				0.104
	0	37	4.60±0.81	4.06±1.10	-0.53±0.90		4.36±0.57	3.92±0.75	-0.43±0.62		4.07±0.60	3.76±0.81	-0.31±0.73		2.26±0.75	2.67±0.78	0.43±0.61	
	1-3	10	4.50±0.77	4.45±0.33	-0.02±0.89		4.27±0.32	4.07±0.57	-0.20±0.55		3.68±0.82	3.76±0.81	.08±0.47		2.70±0.60	2.78±0.68	0.08±0.54	
Anemia						0.890				0.589				0.953				0.020^*^
	0	29	4.63±0.84	4.19±1.09	-0.44±0.76		4.33±0.57	3.98±0.72	-0.34±0.65		4.13±0.54	3.90±0.73	-0.22±0.72		2.27±0.69	2.79±0.72	0.52±0.54	
	1-3	18	4.48±0.71	4.08±0.87	-0.40±1.15		4.35±0.45	3.91±0.71	-0.44±0.54		3.77±0.79	3.53±0.88	-0.23±0.69		2.50±0.81	2.60±0.82	0.10±0.63	
Anorexia						0.018^*^				<0.001^*^				0.007^*^				0.219
	0	27	4.38±0.97	4.23±1.08	-0.15±0.95		4.30±0.57	4.16±0.72	-0.13±0.55		4.00±0.57	4.01±0.69	0.01±0.64		2.21±0.61	2.66±0.68	0.45±0.53	
	1-3	20	4.83±0.30	4.04±0.90	-0.78±0.75		4.40±0.45	3.68±0.60	-0.73±0.51		3.97±0.78	3.43±0.85	-0.54±0.66		2.56±0.86	2.79±0.86	0.23±0.69	

CT: chemotherapy, mean±standard deviation,

*p<0.05

**Table 9:** Comparison of the EQ-5D utility value and QOL-ACD total score according to the presence or absence of anorexia in patients stratified by stage, purpose of chemotherapy and age.

		*n*		EQ-5D			QOL-ACD		
		Grade 0	Grade 1–3	Grade 0	Grade 1–3	*p*	Grade 0	Grade 1–3	*p*
Stage									
	1	6	9	-0.07±0.12	-0.24±0.18	0.061	1.33±5.68	-14.33±10.00	0.004^*^
	2–4	21	11	-0.05±0.17	-0.14±0.12	0.159	1.00±11.34	-7.91±5.49	0.006^*^
Purpose of chemotherapy									
	Neoadjuvant	12	5	-0.10±0.20	-0.07±0.09	0.718	-0.58±14.06	-9.20±9.28	0.231
	Not neoadjuvant	15	15	-0.02±0.11	-0.22±0.15	<0.001^*^	2.40±5.97	-11.33±8.21	<0.001^*^
Age (year)									
	<49	4	7	-0.11±0.13	-0.18±0.11	0.410	4.75±5.19	-11.29±4.61	<0.001^*^
	≥50	23	13	-0.05±0.17	-0.19±0.18	0.021^*^	0.43±10.85	-10.54±9.90	0.005^*^

*p<0.05

## Discussion

In the present study, we measured QOL before and after the first course of outpatient chemotherapy in breast cancer patients using the EQ-5D and QOL-ACD questionnaires. We also investigated adverse events that occurred up until after treatment. This allowed us to assess not only the overall impact of current outpatient chemotherapy-associated adverse events but also the individual impact of each adverse event on QOL in breast cancer patients. The patients were considered representative of the hospital patient population, because all breast cancer patients receiving their first course of chemotherapy in the hospital outpatient setting were enrolled in this study.

Both the EQ-5D utility value and QOL-ACD total score decreased significantly after outpatient chemotherapy in breast cancer patients. According to one report, both the QOL-ACD total score and subscale scores were not different after chemotherapy administration to hospitalized postoperative breast cancer patients [18]. The significant decrease in utility value and QOL-ACD total score after treatment in the present study suggests that the overall QOL in the home and workplace decreases because of outpatient chemotherapy-related adverse events. Moreover, the proportion of patients who responded that they had problems in the mobility and usual activities dimensions of the EQ-5D increased significantly after treatment, whereas the mean scores for the QOL-ACD subscales of activity, physical condition, and psychological condition decreased significantly. This indicates that the adverse events associated with anticancer drugs not only interfere with movement and activity but also exacerbate the patients’ physical and psychological states. On the other hand, the mean score for the QOL-ACD subscale of social relationships increased significantly. The social relationships subscale comprises items on anxiety regarding relationships with family and friends and economic environment. Almost all patients lived with family and therefore, the social aspects of their QOL may have improved because of family support.

Patients with anorexia were found to have a significantly larger decrease in EQ-5D utility value and QOL-ACD total score than patients without anorexia did. In addition, the decrease in the mean score for the QOL-ACD subscales of activity, physical condition, and psychological condition was also significantly greater in patients who experienced this adverse event. This suggests that anorexia has a major impact on QOL. Anorexia is an important prognostic factor for overall survival time [29] and is a concern in patients receiving outpatient chemotherapy [30]. The inability to consume meals causes patients to notice their own illness and feel anxiety, which in turn weakens their will to live. Thus, anorexia may cause particularly strong physical and psychological distress in patients. Therefore, it is very important to control anorexia during initial outpatient chemotherapy sessions in breast cancer patients. For patients receiving neoadjuvant therapy, anorexic patients were found to have a significantly larger decrease in EQ-5D utility value and QOL-ACD total score than non-anorexic patients did. This suggests that anorexia affects on QOL in patients receiving non-neoadjuvant chemotherapy. Patients ≥50 years of age with anorexia were found to have a significantly larger decrease in only QOL-ACD total score than those without anorexia, suggesting that anorexia might influence on QOL in patients ≥50 years of age.

The proportion of patients who responded in the EQ-5D that their ability to perform usual activities had deteriorated after treatment was significantly larger for patients with alopecia, constipation, malaise, and anorexia than for patients without these adverse events. This suggests that many adverse events have a large impact on patients’ usual activities. Moreover, the decrease in the mean QOL-ACD activity subscale scores of patients with fever and anorexia and mean physical condition subscale scores of patients with nausea, oral pain, dysgeusia, and anorexia were significantly larger than that of patients without these adverse events. This indicates that multiple adverse events have an impact on activity and physical condition.

We found that outpatient chemotherapy reduced patients’ overall QOL. Some reports have stated that the QOL of patients receiving outpatient chemotherapy is better than that of patients receiving inpatient chemotherapy [20], whereas other reports have concluded that the QOL of patients receiving inpatient chemotherapy does not change [18]. Because patients receiving outpatient chemotherapy benefit from the care and assistance of their family at home, it is easy to assume that they have sufficient physical and psychological support. However, a considerable number of patients must perform their usual activities at home and the workplace. As a result, our findings indicate that current outpatient chemotherapy for breast cancer may cause multiple adverse events that reduce overall QOL.

In the present study, we assessed the impact of adverse events associated with the first course of outpatient chemotherapy on the QOL of breast cancer patients using the EQ-5D and QOL-ACD questionnaires. Our study had several limitations including small sample size, single institution study, lack of other relevant clinical information, and influence of multiple and lower level adverse events on QOL. Despite these limitations, our findings reveal that adverse events associated with outpatient chemotherapy for breast cancer decrease overall QOL. We also show that anorexia had a particularly large impact on QOL. Therefore, controlling appetite loss during initial outpatient chemotherapy sessions in breast cancer patients is very important. We believe that our study findings are extremely useful and important in understanding the impact of anticancer drug-related adverse events on QOL.
